# Aussie KIDS SAVE LIVES Program: A pilot evaluation of basic life support education in Australian secondary schools

**DOI:** 10.1016/j.resplu.2025.101121

**Published:** 2025-09-30

**Authors:** Natasha Dodge, Joel Marley, Greg Page, Craig Ray, Jason Acworth, Peter T. Morley, Janet E. Bray

**Affiliations:** a School of Public Health and Preventive Medicine, Monash University, Victoria, Australia; b Ambulance Victoria, Victoria, Australia; c Heart of the Nation, New South Wales, Australia; d Australian Resuscitation Council, Victoria, Australia; e Faculty of Medicine, University of Queensland, Brisbane, Australia; f Emergency Department, Queensland Children’s Hospital; g Faculty of Medicine, Dentistry and Health Sciences, The University of Melbourne, Victoria, Australia; h Royal Melbourne Hospital, Victoria, Australia; i Prehospital, Resuscitation and Emergency Care Research Unit, Curtin University, Perth, Western Australia, Australia

**Keywords:** Cardiac arrest, Heart arrest, Cardiopulmonary resuscitation (CPR), Education, Children, Basic life support, KIDS SAVE LIVES

## Abstract

**Introduction:**

Teaching basic life support (BLS) skills to school children has the potential to equip future generations to respond effectively to cardiac arrest. The Aussie KIDS SAVE LIVES pilot program aimed to evaluate the feasibility and usability of teacher-led resources for a 50-minute “Call, Push, Shock” BLS lesson delivered to students in years 7–8 (ages 11–14) in Victoria, Australia.

**Methods:**

Victorian secondary schools were invited to participate in the pilot from November 2022 to December 2023. Teachers and students were invited to complete online evaluation surveys. Descriptive statistics and content analysis were used to assess the effectiveness of the training materials and achievement of the program’s learning objectives.

**Results:**

A total of 56 schools volunteered to participate. Surveys were completed by 25 teachers and 700 students in the pilot period. Teachers reported the training kits and PowerPoint slides as the most useful tools. Students with prior cardiopulmonary resuscitation (CPR) training reported greater confidence in performing CPR (66.8% vs 50.5%, *p* < 0.001). Among students, 97% found the kit easy to use, and 53% said they would share what they learned. Students appreciated the hands-on learning and the opportunity to acquire life-saving skills, although some noted challenges, such as the physical demands of CPR.

**Conclusion:**

The pilot demonstrated that a short, teacher-led BLS session is feasible and well-received. The resources were user-friendly, and the program successfully enhanced students’ confidence and motivation to engage with life-saving skills. Findings also reinforce the value of repeated training which aligns with recommendations for annual CPR education in schools.

## Introduction

Cardiac arrest occurring in the community has high mortality.^1^ Survival more than doubles if the person is given cardiopulmonary resuscitation (CPR)^2^ and quadruples with the addition of public defibrillation.^3^ Unfortunately, most patients experiencing cardiac arrest in Australia are not given these lifesaving treatments,^4^ even though CPR instructions are provided in the emergency call.^5^ The main barriers have been identified as lack of perceived skills and confidence^6^, and training.^7^

Public education in basic life support (BLS) is crucial for saving lives, and teaching children these skills in schools is an effective method to reach future generations.^8^ Globally, training schoolchildren in CPR is now endorsed by the World Health Organisation^9^ and the International Liaison Committee on Resuscitation (ILCOR).^10^, ^11^ The existing Australian curriculum is vague regarding these skills and their delivery.^8^ However, studies have shown that Australian students are supportive of learning BLS training in schools ^12^, and a recent systematic review shows that teachers can be trained to provide effective instruction.^13^ Some regions, despite mandates, have reported low rates of children trained,^14^ and schools are not implementing training due to a lack of support and resources,^15^, ^16^ with calls for research to improve implementation.^17^.

To address these issues, key stakeholders in Australia (Australian Resuscitation Council, Ambulance Victoria, the Victorian Department of Education and Training, the Heart Foundation and Heart of the Nation) have come together, through the Aussie KIDS SAVE LIVES initiative, to provide teachers with the resources needed to provide BLS education.^8^ In this study, we aimed to evaluate our AUSSIE KIDS SAVE LIVES program (Call, Push, Shock education) in a pilot study involving year 7 (aged 11–12 years) secondary school classes in Victoria, Australia.

## Methods

The pilot study was a voluntary program offered to all Secondary Schools in the state of Victoria and evaluated via a voluntary, anonymous online survey that participating teachers and students completed at the end of the teaching session. The study was approved by the Monash University Human Ethics Committee (MUHREC#2023-35754-98066) and the Victorian Department of Education and Training Research Committee.

### Setting

In Victoria, a state in Australia, secondary education is comprised of government, catholic and independent institutions providing education to over one million students across years 7 to 12 (ages 11–18 years). This pilot was conducted in Year 7 classes, which sometimes comprise a composite of Year 7 and Year 8 students and other students may be present from some classes.

### Recruitment and consent

Invitations to schools to participate in the program were issued through a Victorian Department of Education and Training Newsletter and advertised at state educational conferences. School principals were provided with an information sheet and provided written consent for their school to participate. Participating schools delivered the 50-minute Call, Push, Shock CPR training lesson, followed by an anonymous evaluation survey completed by teachers and students. Principals and teachers were provided with an information sheet, and gave written consent to participate. Students were provided with an information sheet, and consent was implied if they completed the survey.

### Intervention

**Fig. 1:**
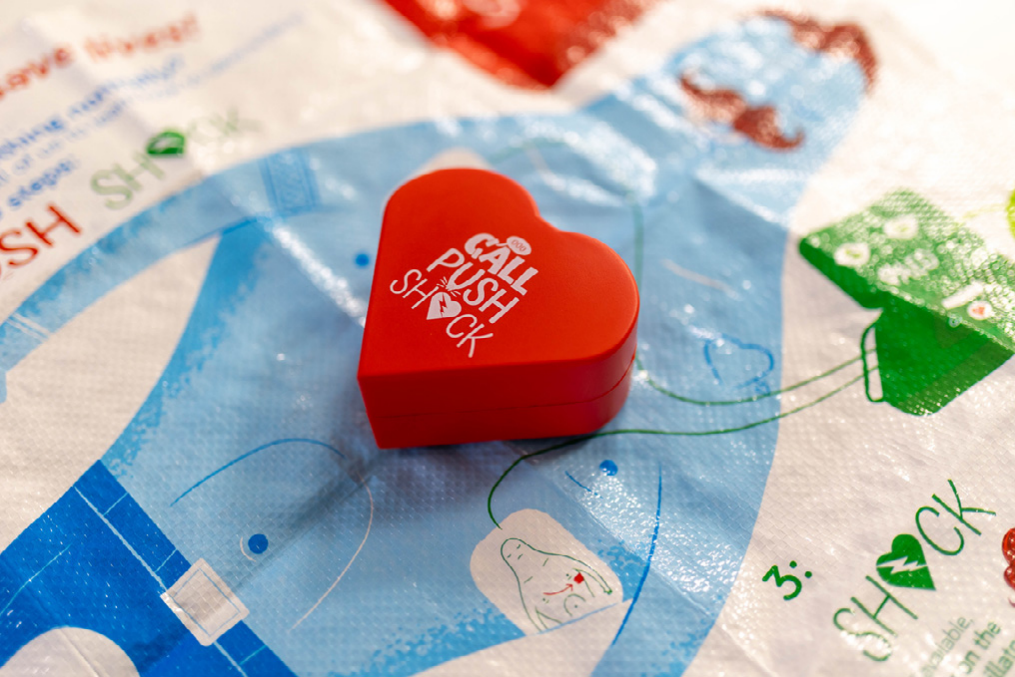
Picture of the Call, Push, Shock training kit.

The Aussie Kids Saves Lives program resources were co-designed with teachers, BLS providers and members of the Department of Education and Training. The 50-minute lesson aimed to educate students on how to save a life in three simple steps (Call, Push, Shock), while also covering other aspects, including recognising cardiac arrest and use of the recovery position. Educational resources provided to teachers included a lesson guide, a curriculum alignment document, a PowerPoint presentation for use in teaching, frequently asked questions and answer sheets, student participation certificates, and a cross-curricular activities document (resources available from the corresponding author). For the pilot, schools were provided with free student training kits, which included a CPR practice mat, automated external defibrillator (AED) instructional information and a squeaking rubber heart to practice chest compressions (Fig. 1).

### Data collection

Teachers and students completed separate anonymous online surveys via Qualtrics (Supplement 1). The surveys were open for completion between November 2022 and December 2023. Surveys were intentionally brief to encourage a high response rate. The student evaluation survey included 10 questions, including three open-ended questions that asked students to reflect on the training session: What did you like the most? What did you like the least? What was the most important thing you learnt? The teacher survey contained a set of closed and open questions used to evaluate the usability and effectiveness of the learning resources provided. Teachers contributed to the development of both surveys, and teachers and a convenience sample of students reviewed the final surveys for face validity.

### Data analysis

Closed-ended questions from the evaluation surveys were analysed using descriptive statistics to summarise participants’ responses. The Chi-squared test was used to explore the impact of prior CPR training on confidence post-training.

The qualitative analysis employed content analysis and was conducted manually in Microsoft Excel. This process began with an initial read-through of all survey responses to develop a general understanding of the data and identify emerging themes. Based on this preliminary analysis, one researcher (ND) developed an initial set of codes to capture key concepts and patterns observed across the responses. Using these initial codes, the data was manually coded. A second researcher (JB) then independently reviewed the survey responses and coding. JB and ND refined the coding framework to ensure clarity, consistency, and comprehensive coverage of the data. The survey responses were subsequently recoded using the revised set of codes to reflect the updated thematic structure and common themes and subthemes identified. Disagreements were resolved by discussion. The researchers involved in the coding and analysis were not involved in the development or delivery of the intervention, and had no contact with the staff or students.

## Results

### Participants

Between November 2022 and December 2023, 56 public and private schools across regional and metropolitan Victoria volunteered to participate in the program. During the survey period, 25 teachers and 700 students completed evaluation surveys.

### Teacher survey results

Of the 25 teachers who completed the survey, all had previous CPR training, and 44% had previously taught BLS or CPR to students (Table 1). When teaching the lesson 84% of teachers used the PowerPoint and 80% used the training kits (Table 2). The majority of teachers (80%) felt confident teaching the lesson and found students to be engaged, with 64% being “very engaged” and 36% being “moderately engaged” (Table 2). The majority of teachers found the training kits to be age-appropriate (92%), and 88% stated that the practice kits were easy to use. The entire lesson plan was covered by 84% of teachers. One teacher said that there was not enough time to practice the recovery position; however, the theory was covered in the lesson. Another teacher noted the class was unable to complete the hand warm-up exercise due to the inappropriate behaviour of students. The lesson schedule for one teacher meant there was insufficient time to include the PowerPoint presentation. Other issues identified were: some teachers reported the noise of the squeaky hearts during compressions was a distraction for students in the lesson and caused disruptions to nearby classes; one teacher said one kit per person would be better than a shared model; and several teachers mentioned they would allow more time for the lesson next time.

**Table 1:** The demographics of the teacher survey respondents (*n* = 25).

**Variable**	**n (%)**
Teaching experience	
0–4 years	7 (28)
5–9 years	4 (16)
10–14 years	2 (8)
15–19 years	5 (20)
20–24 years	2 (8)
25+ years	2 (12)
2 nursing staff	2 (8)

Year level taught*	
Year 7 (ages 11–12)	21 (84)
Year 8 (ages 13–14)	7 (28)
Year 9 (ages 14–15)	2 (8)

How many children did you teach call push shock to?	
0–24	11 (44)
25–49	7 (28)
50–99	4 (16)
100+	1 (4)

Previous BLS training (Yes)	25 (100)

Previously taught BLS or CPR to students (Yes)	11 (44)

BLS: basic life support; CPR: cardiopulmonary resuscitation.

* Multiple responses accepted.

**Table 2:** Responses to the teacher survey after educating (*n* = 25).

**Question**	**n (%)**
Which of the Call, Push, Shock resources did you access to PREPARE for teaching your class?*	
The Call, Push, Shock lesson plan	19 (76)
The frequently asked questions and answers	13 (52)
The Call, Push, Shock Training Kits	23 (92)
The Call, Push, Shock PowerPoint	23 (92)
The Call, Push, Shock Curriculum alignment document	11(44)

Which of the Call, Push, Shock resources did you use when TEACHING your class?*	
The Call, Push, Shock lesson plan	20 (80)
The frequently asked questions and answers	6 (24)
The Call, Push, Shock Training Kits	20 (80)
The Call, Push, Shock PowerPoint	21 (84)
The Call, Push, Shock Curriculum alignment document	2 (8)

**How confident did you feel in teaching the program to the class?**	
Highly confident	20 (80)
Moderately confident	5 (20)
Slightly confident	0 (0)
Not at all confident	0 (0)

**Did you cover the entire lesson plan?**	
Yes	21 (84)
No	4 (16)

**Did your students find the class engaging?**	
Very engaging	16 (64)
Moderately engaging	9 (36)
Somewhat engaging	0 (0)
Not at all engaging	0 (0)

**Were the Call, Push, Shock kits easy to use?**	
Very easy	22 (8)
Moderately easy	3 (12)
Somewhat easy	0 (0)
Not at all easy	0 (0)
Did not use them	0 (0)

**Where the learning materials age appropriate?**	
Yes	23 (92)
No	2 (8)

* Multiple responses accepted.

### Student survey results

**Fig. 2:**
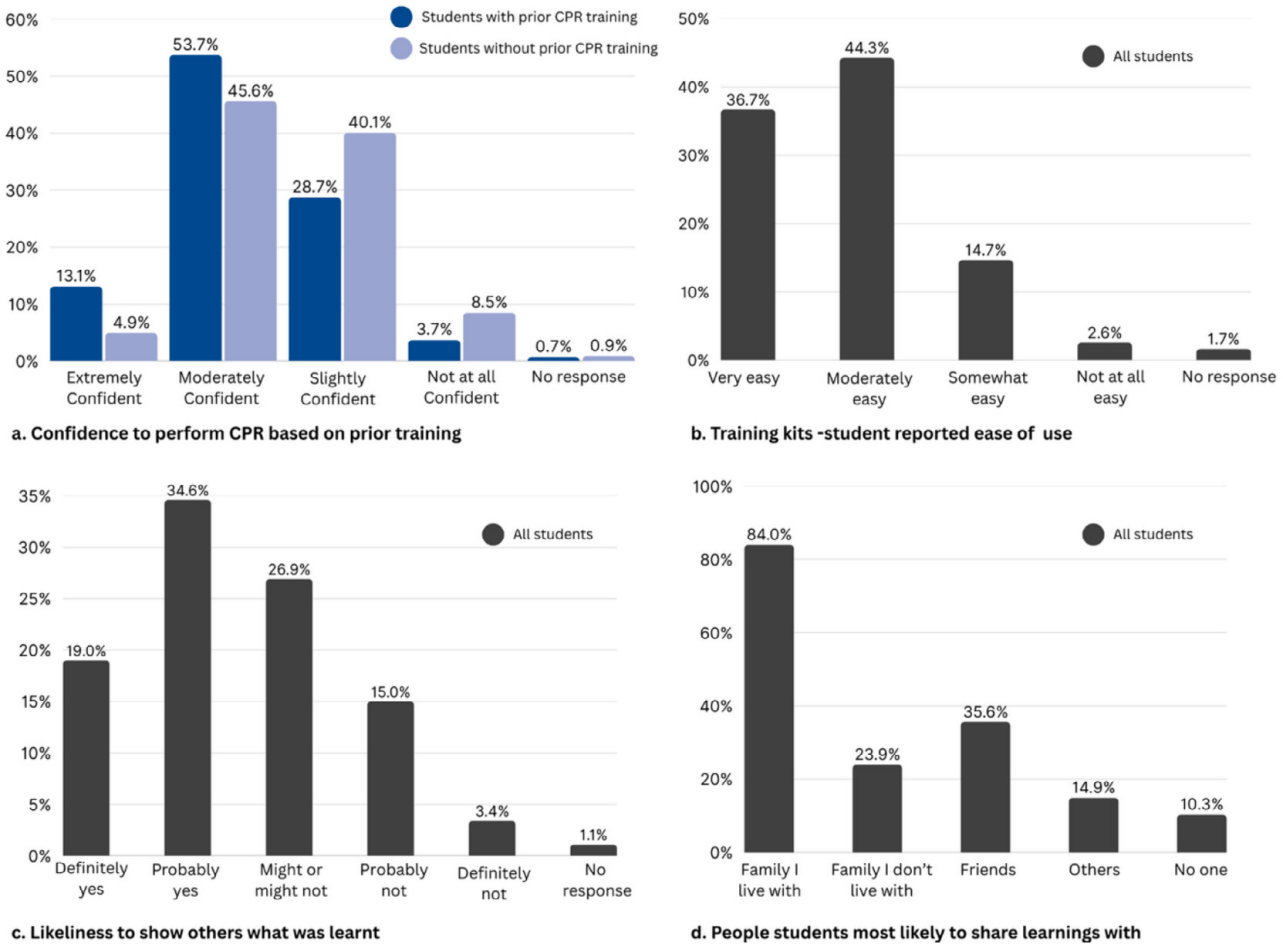
Student responses after training (*n* = 700) for: a. Confidence in knowing how to perform CPR, b. ease of use of training kits, c. likelihood to share learnings, d. who students will share learning with. CPR: cardiopulmonary resuscitation.

The student evaluation survey was completed by 700 students, with 74% (*n* = 516) of responses from students in year 7 (Table 3). Of the students who completed the survey, 38.5% had previously completed CPR training. Students who had previously completed CPR training felt more confident (rated as extremely confident or moderately confident) to provide CPR than those who had no prior training (67% vs 50%) (Fig. 2a). Most students said they found the training kit easy to use (96%) (Fig. 2b). When asked if they would share what they had learnt with someone outside the classroom, 19% said “definitely yes” and 35% said “probably yes” (Fig. 2c). Students were most likely to show the Call, Push, Shock Kit to family members in their home (84%) (Fig. 2d).

**Table 3:** Demographics of student respondents (*n* = 700).

**Demographic variable**	**n (%)**
Year Level	
Year 7 (ages 11–12)	516 (74)
Year 8 (ages 13–14)	148 (21)
Year 9 (ages 14–15)	31 (4)
Year 10 (ages 15–16)	1 (<1)
No response	4 (<1)

Previous CPR training	
Yes	268 (38)
No	329 (47)
Unsure	97 (14)
No response	6 (1)

CPR: cardiopulmonary resuscitation.

The majority of students responded to the open-ended questions. Key themes emerged from these questions: the benefits of hands-on learning and the usefulness of the provided CPR training kits, the physical difficulty associated with performing CPR; and the value of knowing effective CPR and when to use it.

### What they liked most

#### Hands-on learning

The benefits of the training kits and hands-on learning were evident in the response to the question *“What did you like most about the call push shock lesson?”,* where 40% of students stated that they liked the hands-on, practical nature of the lesson:

- *“Being able to practice CPR on something that isn’t real, but teaches you how it can be real”*
- *“I like how we could experience what it would feel like to perform CPR on someone”*
- *“That we got to physically go to try CPR on a dummy that resembles a real body”*

In addition to hands-on learning, many students enjoyed receiving the training kit and using the squeaky heart.

- *“The pack that came along with the lesson.”*
- *“ I liked the squeaky heart”*
- *“ I liked getting our own “Call, Push, Shock” bags that came with the equipment to practice at home and with others.”*

### What was most important

#### Learning lifesaving skills

When asked about the most important thing they learned from the Call, Push, Shock lesson; most students (63%) said that learning effective CPR that could save a life was the most important thing they learned.

- *“The proper position the hands should be in for an effective CPR experience.”*
- *“The most important thing I learnt was how to do CPR effectively.”*
- *“How to deliver chest compressions.”*

Students also believed that learning effective CPR and knowing they can utilise these new skills to save a life was beneficial, with 23% stating it was the thing they liked the most about the lesson.

- *“That I now know how to perform CPR properly, because I never knew how to do it.”*
- *“Learning CPR and learning how to save someone’s life”*

### What they liked least

Overall, students were very positive in their response to the lesson with many believing that it was worthwhile and that there were no aspects that they didn’t enjoy. In response to the question, ***“****what did you like the least about the lesson?”* many students (23%) commented that they enjoyed all aspects of the lesson. However, some students disliked the content or felt that they were unlikely ever to need to use CPR.

- *“nothing, I enjoyed the lesson”*
- *“Nothing, I enjoyed it and it was helpful”*
- *“I think everything was good, nothing bad about it”*
- *“The fact that I am being taught to do CPR when I’ll probably never do it”*
- *“Understanding the basic knowledge, I disliked the repetition”*
- *“Learning about the basics. (I already knew)”*

The physical difficulty of performing CPR for the full two minutes and the hardness of the heart used to perform CPR were the most disliked components by students (20%).

- *“What I liked least was how my hands hurt after trying CPR”*
- *“Practising CPR was quite tiring, as was moving people into the recovery position.”*
- *“I didn’t like how hard the heart was to push.”*

## Discussion

This pilot evaluation of the Aussie KIDS SAVE LIVES program demonstrated strong feasibility and acceptability for delivering BLS training within the Australian school curriculum when schools are equipped with appropriate materials. Feedback from both teachers and students was overwhelmingly positive.

By providing comprehensive, but easy and ready-to-use resources, the program addressed previously reported barriers to implementation, such as a lack of time and capacity to develop suitable materials.^16^, ^17^ Most teachers in our pilot reported feeling confident delivering the lesson and found the content age-appropriate and easy to use. Minor challenges included time limitations and, in some cases, managing student behaviour. Blending learning approaches, such as combining e-learning and a practical component, has been found to be comparable to face-to-face education in learning outcomes,^18^ and provides the additional benefit of standardising content and potentially shortening the time taken in class. This approach may be more suitable for older students.

Teachers also noted high levels of student engagement, highlighting a strong willingness among students to learn life-saving skills. This mirrors findings other research,^17^, ^19^ which reports similarly high enthusiasm for CPR education and its perceived importance. Students in our pilot echoed these sentiments, describing the lesson as empowering and valuable, with many acknowledging the potential to use their skills to save a life.

Among the 700 students who completed the evaluations, the majority found the kits easy to use and appreciated the hands-on learning experience. Some students, however, noted the physical difficulty of delivering compressions − an issue consistent with prior research showing that younger learners may struggle to reach guideline-recommended compression depth.^20^ In response, we adapted the compression hearts to make them slightly softer and are now piloting the program across all secondary school year levels 7–10.

The kits were designed to be reusable and portable, and students expressed a moderate willingness to share what they learned with others. Extending CPR education beyond the classroom, through homework or extracurricular activities, could expand community reach − particularly among underserved populations who may not otherwise access training.

Consistent with previous research,^21^ students reported increased confidence in performing CPR following training, especially those with prior experience. These findings support the value of repeated training and align with ILCOR’s recommendation for annual CPR education in schools.^10^, ^11^ Ongoing collaboration with government partners will be key to sustaining and scaling this initiative nationally.

While CPR training is not specified in the current national or state curricula, we were able to map the Aussie Kids Save Lives lesson into elements of the Victorian Curriculum: Health and Physical Education strands (Personal, Social and Community Health; Personal and Social Capability) at schools years 7 though 10. It addresses learning outcomes related to recognising and managing emotions, resilience, teamwork and collaboration and health-related decision-making. The teachers appreciated this additional effort and this may have contributed to the high uptake of the program. The AUSSIE KIDS SAVE LIVES sustainability is enhanced by our train-the-trainer approach and by the early involvement of teachers in the development of the teaching materials.^17^ Other researchers have tested integrating technology (e.g. virtual reality training) and this has resulted in high confidence and willingness to perform CPR on others.^22^

### Limitations

This study has several limitations. First, as participation was voluntary, schools implemented the program flexibly within their existing schedules. Although recruitment to the program was high, the surveys were anonymous, and we did not have permission to contact schools directly. As a result, we were unable to determine the exact survey response rate or whether participating schools differed from those that did not participate. Also, some schools were recruited late and may have delivered training outside the designated pilot period. Second, we did not collect data on knowledge or skill acquisition, given that extensive prior evidence has shown that the training modality has little effect on learning outcomes.^21^ Additionally, self-selection bias may have influenced responses, as more engaged teachers and students may have been more likely to complete the survey. While participating schools varied in location, type, and size (data not shown), generalisability should be interpreted with caution.

### Conclusion

This pilot highlights the feasibility of equipping secondary school students with lifesaving CPR skills through a scalable, low-cost program. Embedding CPR training into the national curriculum, supported by practical, student-centred materials, has the potential to increase the number of capable bystanders prepared to respond during cardiac arrest. To ensure equitable and consistent access, CPR education should be a formal component of the school curriculum, rather than offered as an optional or ad hoc program. This would support long-term skill development and help normalise CPR as a core competency for all students.

## CRediT authorship contribution statement

**Natasha Dodge:** Writing – original draft, Formal analysis, Data curation, Conceptualization. **Joel Marley:** Writing – review & editing, Resources, Methodology, Funding acquisition, Conceptualization. **Greg Page:** Writing – review & editing, Resources, Funding acquisition, Conceptualization. **Craig Ray:** Writing – review & editing, Funding acquisition, Conceptualization. **Jason Acworth:** Writing – review & editing, Funding acquisition, Conceptualization. **Peter T. Morley:** Writing – review & editing, Funding acquisition, Conceptualization. **Janet E. Bray:** Writing – original draft, Project administration, Methodology, Funding acquisition, Formal analysis, Data curation, Conceptualization.

## Declaration of competing interest

JEB and PM are Editors for Resuscitation Plus. Ambulance Victoria sells the training kits used in the study.
